# Age moderates the effect of socioeconomic status on physical activity level among south Korean adults: cross-sectional analysis of nationally representative sample

**DOI:** 10.1186/s12889-019-7610-7

**Published:** 2019-10-22

**Authors:** Harold H. Lee, Ashley E. Pérez, Don Operario

**Affiliations:** 1 Department of Social and Behavioral Sciences, Harvard T.H. Chan School of Public Health, 401 Park Dr., 428F, Boston, MA 02115 USA; 2 Department of Social and Behavioral Sciences, University of California San Francisco School of Nursing, 3333 California St., Suite 455, San Francisco, CA 94118 USA; 3 Department of Behavioral and Social Sciences, Brown University School of Public Health, Box G-S121-3, Providence, RI 02912 USA

**Keywords:** Physical activity, Health disparities, South Korea, Noncommunicable disease

## Abstract

**Background:**

In a nationally representative sample of South Korean adults, we investigated the association between socioeconomic status (SES) and physical activity level, and whether this association varied by age group.

**Methods:**

We used data from 5065 subjects aged ≥19 years who participated in the 6th Korea National Health and Nutrition Examination Survey. Weighted logistic regression examined the SES-physical activity association. Using the International Physical Activity Questionnaire short form, physical activity level was categorized into two groups: meeting the guideline (≥150 min/week of moderate intensity physical activity) and not meeting the guideline. Annual household income quartile (first quartile = highest income) and education (elementary, middle school, high school, and college graduates) were used as SES indicators. Sociodemographic covariates included in the adjusted models were marital status, town type (rural/urban), dwelling type, perceived health, federal allowance support (yes/no), and working- and sleeping-hours.

**Results:**

In unadjusted models, low income and low education were both associated with significantly lower odds of meeting the physical activity guideline. The income-physical activity association was moderated by age group in both unadjusted and adjusted models. Specifically, among those age < 45 years, those in the third quartile group had 41% higher odds (*p* < 0.01) of meeting the physical activity guideline compared with the first quartile group, after adjusting for covariates. In contrast, among those age ≥ 45 years, those in the third quartile group had 16% lower odds (p < 0.01) of meeting the physical activity guideline compared with the first quartile group, after adjusting for covariates. Furthermore, the education-physical activity association was moderated by age but only in the adjusted model. Specifically, among those age < 45 years, high school graduates had a 21% higher odds of meeting the physical activity guideline compared with college graduates (*p* = 0.08). In contrast, among those age ≥ 45 years, high school graduates had a 23% lower odds of meeting the physical activity guideline compared with college graduates (*p* = 0.01).

**Conclusion:**

Future policies that aim to address SES-related health disparities in physical activity among adults in South Korea should consider the different patterns of physical activity in accordance with SES and age.

## Background

South Korea’s economy grew rapidly over the last 40–45 years, achieving a 7% growth rate per year in real per capita income [1]. This exceptional economic growth, however, has been accompanied by increasing income and educational inequalities [2, 3]. More recently, there is an emerging concern about socioeconomic disparities in overall mortality as well as disparities in the prevalence of noncommunicable diseases (NCDs) such as diabetes, hypertension, and obesity among South Koreans [4–8].

One of the four major modifiable behavioral risk factors for NCDs, along with diet, smoking, and drinking, is low rate of physical activity, which accounts for ~ 9% (5.3 million) of premature deaths globally [9]. To curb this global problem, the World Health Organization (WHO) recommended that nations develop physical activity action plans and policies to increase physical activity levels in their populations [10]. Specifically, WHO recommended that adults engage 150 min per week of moderate-intensity physical activity (e.g., brisk walking) or 75 min per week of vigorous physical activity (e.g., running), or an equivalent combination of moderate- and vigorous-intensity aerobic activity [11]. According to the WHO Global Health Observatory data repository, the proportion of adults meeting this recommendation is lower in high-income compared to low-income countries [12, 13], with most adults from developed countries (e.g., Australia, UK, and US) not meeting the recommendations [14–16]. In this light, among the OECD countries, following Mexico and Costa Rica, South Korea is ranked 3rd in terms of hours spent on working (i.e., 2024 h per week in 2017) [17], which may minimize the time and cognitive energy to engage in health promoting behaviors, including sleeping and physical activity [18, 19]. Furthermore, a corpus of research conducted in high-income countries has shown that socioeconomically disadvantaged populations are even less likely to engage in adequate levels of physical activity compared with those who have greater socioeconomic advantage [20–25].

Given emerging evidence of health disparities in South Korea, as well as the unequal distribution of meeting physical activity guidelines by socioeconomic status (SES) observed in other high-income countries, low SES Korean adults may be at risk for engaging in lower physical activity levels. While a few studies to date reported that low SES is associated with lower participation in physical activity among adolescents [26], the elderly [27], and metabolic syndrome patients [28] in South Korea, to our knowledge, no studies have examined this relationship among healthy South Korean adults (age ≥ 19). To address this gap, the specific objective of the present study was to investigate the associations between two SES indicators, annual household income and education, and physical activity in a nationally representative sample of South Korean adults. We hypothesized that low household income and education status would be associated with lower odds of meeting the WHO physical activity guideline. However, previous literature has reported that physical activity tends to decline as age increases [12]; specifically, a more progressive decline in physical activity is observed after age 45 [16, 29]. Therefore, we further explored whether age group (with age 45 as the cut point) moderated the relationship between SES and physical activity participation.

## Methods

### Overview

**Fig. 1:**
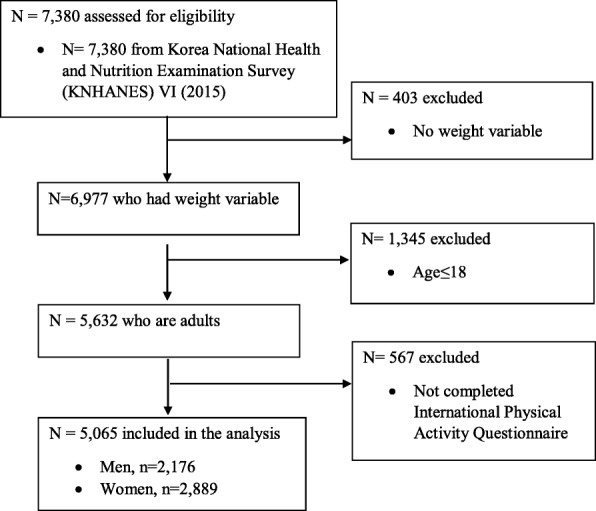
Flow diagram of subject inclusion and exclusion in KNHANES 2015

The present study employed a cross sectional design using nationally representative data from South Korea. A total of 7380 subjects participated in the 6th Korea National Health and Nutrition Examination Survey (KNHANES VI; 2015) conducted by the Korean Centers for Disease Control and Prevention (KCDC) and the Ministry of Health and Welfare. A full description of the KNHANES methodology has been presented elsewhere [30, 31]. Briefly, KNHANES is a nationally representative sample of non-institutionalized South Koreans. In order to recruit a representative sample, KNHANES utilized a multi-stage clustered probability design based on administrative district, place of residence, and residential means (i.e. apartment, other than apartment). Participants completed in-person health interviews and health examinations in mobile examination centers with trained staff members. From the total 7380 subjects, we excluded 2315 participants for the following reasons: missing weight variables, aged below 19 years old, and did not completed the International Physical Activity Questionnaire (IPAQ) (Fig. 1). Our analytic sample included the remaining 5065 subjects who completed the IPAQ. However, some covariates had missing values: ‘Household income’ was missing among 22 subjects (0.7%), ‘Education’ was missing among 9 subjects (0.1%), ‘Federal allowance support’ was missing among 4 subjects (0.2%), ‘Sleeping hours’ was missing among 74 subjects (1.4%), ‘Perceived health’ was missing among 2 subjects (0.01%), and ‘Working hours’ was missing among 1642 subjects (32.4%). We considered removing working hours given the high proportion of missing data, but working hours is a plausible confounder of the SES-PA link so we decided to include it. Overall, relative to those who provided IPAQ data, those who did not were more frequently married, older, and had a lower education (*p* < 0.05).

### Measures

In accordance with other studies on SES gradients in health [32], we examined two self-reported SES indicators included in the KNHANES: annual household income and education. Household income was assessed by dividing monthly household income by the square root of the number of household members (adjusting for sex and each 5-year age stratum), and participants were categorized into four quartile household income groups (upper, moderate, moderate-low, and low). Education was assessed by the question, “What is the highest diploma you obtained from school?” (categories included college graduate, high school graduate, middle school graduate, and elementary school graduate). The primary dependent variable used for this analysis was total volume of physical activity, which was computed using the IPAQ short form. The 9-item IPAQ questionnaire assesses the amount of time spent engaging in five-levels of weekly physical activity: 1) vigorous physical activity spent during work, 2) moderate physical activity spent during work, 3) vigorous physical activity spent during leisure time, 4) moderate physical activity spent during leisure time, and 5) moderate physical activity spent on transportation. To calculate total volume of moderate intensity physical activity, KNHANES staff members doubled the time spent in vigorous physical activity (e.g., 30 min of vigorous physical activity = 60 min of moderate intensity physical activity). Then, all minutes were added. KNHANES staff then categorized subjects into two groups in a variable called ‘pa_aerobic’: those who do not meet the WHO physical activity guideline (≤150 min per week of moderate-intensity physical activity) and those who meet the physical activity guideline (> 150 min per week of moderate-intensity physical activity) [11]. The Korean version of the short-form IPAQ employed in KNHANES has been shown to be valid and reliable [33, 34]. Additional co-variates included marital status, town type (rural/urban), dwelling type, perceived health, federal allowance support (yes/no), and working- and sleeping-hours.

### Analytic strategy

All analyses were conducted using R [35], and weights were applied using the *svy* command. First, we conducted descriptive analyses to examine distributions for all key variables; weighted percentages are reported here. Second, we examined bivariate associations between SES indicators and our primary dependent variable, physical activity. Third, we employed weighted logistic regressions to examine the association between each socioeconomic factor and physical activity level, adjusting for sociodemographic covariates including marital status, town type (rural/urban), dwelling type, perceived health, federal allowance support (yes/no), and working- and sleeping-hours. Given limited research on SES and physical activity in South Korea, covariates were selected as potentially intermediary or confounding variables rather than based on prior literature. Finally, we examined interactions between SES and age group (age < 45 vs. age ≥ 45), adjusting for sociodemographic covariates.

## Results

Sociodemographic characteristics of the 5065 participants are shown in Table 1 (unweighted and weighted percentages). The mean age was 43.6 (SE = 0.4) years. About one-quarter of adults aged 19 and older, 76.4% were married, and 75.1% had graduated high school. Overall, 49.0% were classified as not meeting the WHO physical activity guideline.

**Table 1:** Population-Weighted Characteristics of the KNHANES VI Study Sample (*n* = 5065)

	n (weighted %)
Age, mean (SE)	43.6 yr (0.38)
Age ≥ 45 years	3269 (52.5%)
Female	2889 (51.4%)
Married	4240 (76.4%)
Federal allowance support	4656 (92.5%)
Working hours, mean (SE)	41.20 h/wk. (0.39)
Sleeping hours, mean (SE)	6.76 h/d (0.02)
Town type	
Dong (i.e., Urban)	4113 (83.6%)
Ub or myun (i.e., Rural)	948 (16.4%)
Dwelling	
Single house	1860 (35.6%)
5-story apartment and higher	2541 (50.2%)
4-story apartment and lower (> 660 ft^2^)	362 (7.1%)
4-story apartment and lower (≤660 ft^2^)	226 (4.5%)
Other	76 (1.5%)
Perceived health	
Very good	245 (5.4%)
Good	1241 (25.8%)
Normal	2549 (50.5%)
Bad	812 (14.9%)
Very bad	216 (3.4%)
Household income	
1st quartile	1464 (31.3%)
2nd quartile	1381 (30.1%)
3rd quartile	1240 (23.3%)
4th quartile	958 (15.3%)
Education	
College graduate	1623 (37.6%)
High school graduate	1705 (37.5%)
Middle school graduate	549 (8.7%)
Elementary school graduate	1179 (16.3%)
Meeting the physical activity guideline	
Yes	2374 (51.0%)
No	2691 (49.0%)

Bivariate associations of SES and physical activity by age and gender are illustrated in Table 2. Weighted Chi-square statistics showed that the probability of meeting the physical activity guideline significantly differed by income and education. With regard to income, the proportion of those meeting the physical activity guideline was comparable at upper (54.7%), moderate (52.8%), and moderate-low (50.0%) income levels, but lower among those in the low income group (41.1%), F (1, 3) = 34.10, *p* < 0.001. With regard to education, the proportion of those meeting the physical activity guideline was comparable for college graduates (55.7%) and high school graduates (55.7%), lower among middle school graduates (43.2%), and lowest among those who completed elementary school or below (33.5%), F (1, 3) = 7.20, *p* < 0.001.

**Table 2:** Habitual Physical Activity Level by Two SES Indicators; KNHANES VI (n = 5065)

Household income	Upper	Moderate	Moderate-low	Low	test statistics	*p*-value
Meeting PA guideline	54.7%	52.8%	50.0%	41.1%	F = 34.10	< 0.001
Not meeting PA guideline	45.3%	47.2%	50.0%	58.9%		
Education	College graduate and above	High school graduate	Middle school graduate	Elementary school graduate and below		
Meeting PA guideline	55.7%	55.7%	43.2%	33.5%	F = 7.20	< 0.001
Not meeting PA guideline	44.3%	43.9%	48.4%	57.2%		

Comparison of SES and physical activity categories: Weighted Chi-square

The unadjusted and adjusted weighted logistic regressions testing associations of SES and SES*age on physical activity are shown in Table 3. In the unadjusted model, education and income were both significant predictors of meeting the physical activity guideline. Specifically, compared to the upper household income group, the moderate-low- (OR = 0.83, 95% CI: 0.69–0.99) and low household income groups (OR = 0.58, 95% CI: 0.47–0.71) had significantly lower odds of meeting the physical activity guideline. Additionally, compared to college graduates, middle school (OR = 0.61, 95% CI: 0.48–0.71) and elementary school graduates (OR = 0.40, 95% CI: 0.33–0.49) had significantly lower odds of meeting the physical activity guideline. Age was a significant predictor of meeting the physical activity guideline in the unadjusted model. Specifically, older adults (age ≥ 45 years) had a significantly lower odds (OR = 0.54; 95% CI: 0.46–0.62) of meeting the physical activity guideline compared with younger adults.

**Table 3:** Weighted Logistic Regressions examining Associations between Socioeconomic Status and Age with Physical Activity; KNHANES, VI (n = 5065)

	Unadjusted			Fully Adjusted ^a^		
Model 1	OR	CI	p-value	Adjusted OR	CI	p-value
Household Income						
Upper	1.00	n/a	n/a	1.00	n/a	n/a
Moderate	0.92	0.75–1.14	0.47	1.01	0.80–1.29	0.89
Moderate-low	**0.83**	**0.69–0.99**	**0.04**	1.08	0.87–1.35	0.47
Low	**0.58**	**0.47–0.71**	**< 0.01**	0.80	0.59–1.10	0.17
Education						
College graduate	1.00	n/a	n/a	1.00	n/a	n/a
High school graduate	1.00	0.85–1.19	0.99	1.12	0.91–1.36	0.28
Middle school graduate	**0.61**	**0.48–0.77**	**< 0.01**	0.83	0.62–1.11	0.21
Elementary school graduate	**0.40**	**0.33–0.49**	**< 0.01**	0.70	0.52–0.93	0.02
Age						
Younger (Age < 45)	1.00	n/a	n/a	1.00	n/a	n/a
Older (Age ≥ 45)	**0.54**	**0.46–0.62**	**< 0.01**	**0.75**	**0.63–0.89**	**< 0.01**
Model 2: SES*Age Interaction	OR	CI	p-value	Adjusted OR	CI	p-value
Household Income						
Younger upper	1	n/a	n/a	1.00	n/a	n/a
Younger moderate	1.28	1.11–1.48	0.08	1.15	0.84–1.58	0.38
Younger moderate-low	1.21	0.97–1.51	0.79	**1.41**	**1.02–1.97**	**0.04**
Younger low	0.92	0.42–0.94	0.3	1.10	0.57–2.13	0.77
Older upper	0.93	0.75–1.16	0.55	1.22	0.90–1.66	0.20
Older moderate	**0.67**	**0.49–0.91**	**0.01**	1.07	0.88–1.30	0.19
Older moderate-low	0.67	0.30–1.53	0.34	**1.02**	**0.88–1.18**	**0.01**
Older low	0.76	0.24–2.43	0.65	0.79	0.44–1.42	0.15
Education						
Younger college graduate	1.00	n/a	n/a	1.00	n/a	n/a
Younger high school graduate	1.21	0.97–1.51	0.08	1.14	0.88–1.47	0.34
Younger middle school graduate	0.90	0.42–1.94	0.79	1.59	0.63–3.98	0.32
Younger elementary school graduate	0.54	0.17–1.74	0.30	0.76	0.19–3.07	0.70
Older college graduate	0.93	0.75–1.16	0.55	1.08	0.82–1.42	0.57
Older high school graduate	**0.77**	**0.65–0.89**	**0.01**	1.15	0.39–1.57	0.73
Older middle school graduate	0.57	0.20–1.59	0.35	0.81	0.54–1.18	0.13
Older elementary school graduate	0.39	0.01–15.07	0.65	0.73	0.01–45.6	0.86

^a^ Models controlled for marital status, town type, dwelling type, perceived health, federal allowance support, and working- and sleeping-hours

Statistically significant data (p<0.05) are presented in bold

**Fig. 2:**
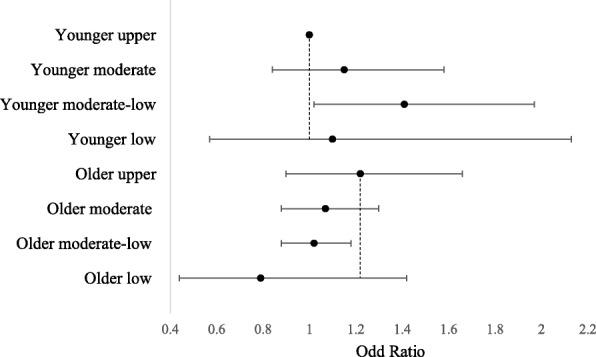
Household Income and Odds of meeting the Physical Activity Guideline by Age, Adjusting for Covariates

We observed two significant interaction effects (i.e., income × age; education × age; *p* < 0.01) (Table 3). First, in unadjusted and adjusted models, the association between income and physical activity was moderated by age group (Table 3, Model 2; also shown in Fig. 2). Specifically, after adjusting for sociodemographic covariates, younger adults (age < 45 years) in the moderate-low household income group had a 41% higher odds of meeting the physical activity guideline compared with younger adults in the upper household income group (OR = 1.41, 95%CI = 1.02–1.97; *p* = 0.04). In contrast, among older adults (age ≥ 45 years) the odds of meeting the physical activity guideline decreased as household income decreased. Specifically, compared with the upper household income group, older adults in moderate-low household income had a 16% decreased odds of meeting the physical activity guideline (OR = 0.84, 95%CI = 0.72–0.96; *p* = 0.01), after adjusting for sociodemographic covariates (Fig. 2).

The second significant interaction effect showed that the association between education and physical activity was moderated by age in the unadjusted model (Table 3, Model 2). Specifically, among younger adults (age < 45 years), high school graduates had a 21% higher odds of meeting the physical activity guideline compared with college graduates (OR = 1.21, 95%CI = 0.97–1.51; *p* = 0.08). By contrast, among older adults, high school graduates had 23% lower odds of meeting the physical activity guideline compared with college graduates (OR = 0.77, 95%CI = 0.65–0.89; *p* = 0.01). The moderating effect was no longer significant after adjusting for covariates (*p* > 0.05).

## Discussion

In this nationally representative study, we observed that 51% of adults in South Korea were meeting the physical activity guideline recommended by WHO. This estimate, which was computed using IPAQ, is similar to those reported in the US (52%) from Behavioral Risk Factor Surveillance System data [13] and lower than Japan (66.2%), Singapore (66.9%), and China (75.9%) from WHO’s estimation using the Global Physical Activity Questionnaire [36]. Altogether, these indicate that adults in South Korea may be physically inactive compared to other high-income countries in Asia. However, prior research has shown that IPAQ and the Global Physical Activity Questionnaire both tend to show a trend of over-reporting, which in turn leads to an underestimation of physical inactivity prevalence [37–40]. In fact, a pooled analysis involving 358 population-based surveys with 1.9 million participants that adjusted for this over-report suggested a much lower prevalence (27.5%) of meeting the guideline [41]. Taken together, given the variability in measures assessing physical activity, comparison between countries should be made with caution. Based on a more detailed analysis, this trend in South Korea varied by SES characteristics, such that low income and low educational attainment were associated with lower physical activity—thereby suggesting potential health consequences related to low physical activity levels in these low SES groups. To our knowledge, this is the first study to demonstrate such disparities in physical activity behavior among adults in South Korea.

The associations between SES and physical activity level were complex. Specifically, we observed that age moderated the association between income and physical activity, such that younger adults with moderate-low income levels were more physically active than younger adults with high-income levels. In contrast, physical activity levels of older adults showed a reverse trend, such that physical activity levels declined among lower income groups. A similar trend was observed with education, but only in the unadjusted model, which highlights the possibility that income is a more proximal correlate of physical activity behavior than education. The specific mechanisms explaining these associations remain unclear. The effect modification of the SES-physical activity association by age can be interpreted with emphasis on the South Korean context. In South Korea, more than 45% adults who are in poverty are over 65 years old, and this is the highest rate among the 38 OECD countries [42]. In addition, there may be a cohort effect such that the current elderly generations were alienated from the formal education system when they were school age, due to the Korean War is the 1950s. In light of this specific national context, the SES*age interaction patterns found here warrant examination in other settings to determine generalizability. However, there is compelling evidence of decline in muscle mass with increasing age [43], which in part accounts for lower physical activity among older adults. One potential explanation for the trend observed among younger adults could be that younger adults at high income levels live more sedentary lives due to their occupations (e.g., office work, managerial positions) as well as due to amenities (e.g., private transportation, larger home dwellings) that permit reduced levels of daily physical exertion, whereas younger adults with more modest income levels might have jobs or social contexts that require greater levels of daily activity (e.g., physically laborious jobs, walking/biking for transportation). The association between reduced physical activity and income among older adults might reflect a different social explanation—i.e., that older adults with higher income levels have access to resources and environments that encourage physical activity for recreation (e.g., gyms and sports clubs), whereas older adults with lower income levels lack these conditions that permit physical recreation. Further research is necessary to examine the specific factors that might explain these physical activity disparities in accordance with income and age in South Korea.

In light of the growing health disparities in Korea, these moderating effects warrant future investigation for hypothesis-driven social science research as well as evidenced-based policy implementation. For example, interventions that promote access to recreational activities (e.g., sports teams or clubs) might be effective at promoting physical activity for older adults with low income and education levels, who might lack these resources and for whom physical activity might not be seen as enjoyable or rewarding. In contrast, interventions that caution against sedentary lifestyles might be effective at promoting physical activity for high-income younger adults, for whom the demands of work and the use of modern amenities might render physical activity as a low priority.

More broadly, one of the major policy implications from this work is that South Korea should conduct regular surveillance of physical activity. This data could be used, in turn, by public health scientists and policy makers to identify correlates and determinants of physical activity that should be targeted via intervention, policy, and campaigns. Another policy implication is that KNHANES may consider adding the Global Physical Activity Questionnaire to the existing IPAQ so as to facilitate a valid between-country comparison.

There were several strengths of the present study. We examined nationally representative data using weighted analysis, which permits generalization of findings to South Korean adults. Therefore, the observations from the present study have high external validity. Moreover, the IPAQ is a well-validated, reliable prognostic indicator of clinical health outcomes that can effectively discriminate between populations [44–46]. Finally, while prior studies examined the association between SES and physical activity among adolescents [26], the elderly [27], and metabolic syndrome patients [28] in South Korea [26], this is the first study to examine the association among adults in South Korea and is an important step toward understanding population patterns associated with physical activity in this national context.

There were also several limitations of the present study. We cannot infer the causal effect of SES on physical activity because we employed a cross-sectional design. Reverse causation is theoretically plausible, such that physical activity level may influence household income and academic achievements. However, this scenario seems unlikely considering existing empirical research and social science theory that highlights the effect of SES on health behavior in general [32, 47] and physical activity in particular [20–25], rather than the reverse pathway. There may be limitations to using household income for age-specific comparisons. For example, using household income assumes that all family members benefit equally from the household income, which may not be the case. Finally, physical activity was measured using a self-report questionnaire, which is susceptible to biases [48]. However, the Korean version of IPAQ has been shown to be valid and reliable [33, 34].

## Conclusion

Overall, roughly half of adults in South Korea are physically inactive, which indicates a high proportion of inactivity in comparison with adults in other high-income national settings. Consistent with growing research on socioeconomic disparities in Korea, the proportion meeting the WHO recommendation for physical activity—which is an important behavior that can promote overall health and wellbeing—was disproportionately higher among wealthy and educated adults in Korea. Future policies that aim to address physical activity-related health disparities in Korea must incorporate different patterns of physical activity in accordance with SES and age. Specifically, a more tailored intervention or policy approach may be required to address lower physical activity levels among younger adults with higher income and among older adults with lower income. A tailored approach among older adults with lower income appears to have particularly high public health relevance given the rapidly aging population in South Korea.

## Data Availability

The Korea National Health and Nutrition Examination Survey (KNHANES) data utilized for the present article is publicly available through the KNHANES website (http://knhanes.cdc.go.kr). The website is only available in Korean. In the website, click “원시자료다운로드”. Then, enter an email address to receive the data.

## Abbreviations

IPAQ: International Physical Activity Questionnaire
KNHANES: Korea National Health and Nutrition Examination Survey
NCDs: Noncommunicable diseases
SES: Socioeconomic status
WHO: World Health Organization
